# Evaluation on the Efficacy and Immunogenicity of Recombinant DNA Plasmids Expressing Spike Genes from Porcine Transmissible Gastroenteritis Virus and Porcine Epidemic Diarrhea Virus

**DOI:** 10.1371/journal.pone.0057468

**Published:** 2013-03-19

**Authors:** Fandan Meng, Yudong Ren, Siqingaowa Suo, Xuejiao Sun, Xunliang Li, Pengchong Li, Wei Yang, Guangxing Li, Lu Li, Christel Schwegmann-Wessels, Georg Herrler, Xiaofeng Ren

**Affiliations:** 1 College of Veterinary Medicine, Northeast Agricultural University, Harbin, China; 2 College of Life Sciences, Northeast Agricultural University, Harbin, China; 3 Institute of Virology, University of Veterinary Medicine, Hannover, Germany; Federal University of Pelotas, Brazil

## Abstract

Porcine transmissible gastroenteritis virus (TGEV) and porcine epidemic diarrhea virus (PDEV) can cause severe diarrhea in pigs. Development of effective vaccines against TGEV and PEDV is one of important prevention measures. The spike (S) protein is the surface glycoprotein of TGEV and PEDV, which can induce specific neutralization antibodies and is a candidate antigen for vaccination attempts. In this study, the open reading frames of the TGEV S1 protein and in addition of the S or S1 proteins of PEDV were inserted into the eukaryotic expression vector, pIRES, resulting in recombinant plasmids, pIRES-(TGEV-S1-PEDV-S1) and pIRES-(TGEV-S1-PEDV-S). Subsequently, 6–8 weeks old Kunming mice were inoculated with both DNA plasmids. Lymphocyte proliferation assay, virus neutralization assay, IFN-γ assay and CTL activity assay were performed. TGEV/PEDV specific antibody responses as well as kinetic changes of T lymphocyte subgroups of the immunized mice were analyzed. The results showed that the recombinant DNA plasmids increased the proliferation of T lymphocytes and the number of CD4+ and CD8+ T lymphocyte subgroups. In addition, the DNA vaccines induced a high level of IFN-γ in the immunized mice. The specific CTL activity in the pIRES-(TGEV-S1-PEDV-S) group became significant at 42 days post-immunization. At 35 days post-immunization, the recombinant DNA plasmids bearing full-length S genes of TGEV and PEDV stimulated higher levels of specific antibodies and neutralizing antibodies in immunized mice.

## Introduction

Transmissible gastroenteritis (TGE) and porcine epidemic diarrhea (PED) are both severe enteric diseases in newborn piglets which are characterized by extremely high mortality, as well as by devastating economic consequences for swine industry [3], [32], [35]. The etiologic agents responsible for these diseases are coronaviruses, TGEV and PEDV, respectively. TGEV was isolated for the first time in 1946 [8]. Japan and England reported the disease in 1956 and 1957 [12], [31]. The virus replicates in the cytoplasm of mature absorptive epithelial cells present on the tips of the villi in the small intestine. The functions of the coronavirus spike (S) protein are both attachment to the cell surface and fusion of the viral membrane with the cellular membrane [7], [36]. The S protein is the major inducer of TGEV-neutralizing antibodies [11], [15], [19]. Therefore, it is an excellent target protein candidate for vaccine development. The relevant epitopes for neutralization were mapped to the N-terminal domain of S protein, and four antigenic sites (A to D) were identified within the first 543 of the 1447 residues of the S protein [13], [20]. The first 37% of the polypeptide chain of the S protein appear to be more immunogenic than the rest of the sequence. This region would be located in the globular part of the peplomer, which is more exposed than the fibrillar, C-terminal portion of the S protein [13]. Previous reports show that the immunogenicity of the DNA vaccine comprising the main antigenic sites is superior to a vaccine containing the total length S gene [29].

PEDV is related to TGEV and bears similarities in its structure as well as in the clinical disease and lesions induced [1], [9]. PEDV was first separated in Belgium and the United Kingdom in 1978 [2], [28], [47]. The disease is characterized by severe diarrhea, vomiting, dehydration, and death, and has a mortality rate of up to 90% [35]. Since 1978, the disease has frequently broken out in many swine-raising countries and has resulted in severe economic losses in Asia, notably in China, Japan and Korea [6], [14], [18]. In 1996, PED outbreaks have been reported to be responsible for the death of more than 39,000 piglets in Japan [42]. PED caused not only the death of neonatal piglets, but also the weight loss in fattening pigs due to PEDV-induced diarrhea. Therefore, it is important to develop an effective vaccine preventing PEDV infection.

The PEDV S protein also plays an important role in induction of neutralizing antibodies, specific receptor binding and cell membrane fusion [10]. The S protein is not cleaved into S1 and S2 subunits by furin-like proteases, due to the lack of appropriate cleavage sites. The S1 domain (residues 1–789) and the S2 domain are artificially defined on the S protein (residues 790–1.383) [10], [34]. Previous reports have shown that the main neutralizing epitopes are located on the S1 domain that is thought to form the globular part of S protein [34], [39]. Sun et al. (2007) reported that the epitope region designated S1D (aa 636∼789) on the S1 domain of PEDV S protein is highly conserved across PEDV isolates and that this region has the capacity to induce the production of virus neutralization antibodies. Moreover, the immune serum against S1D showed the binding ability to the native S protein of PEDV. The S1D5 (aa 744–759) and S1D6 (aa 756–771) are two linear epitope domains. Furthermore, the SS2 (-748 YSNIGVCK 755-) and SS6 (-764 LQDGQVKI 771-) are two core epitope domains on S1D5 and S1D6, respectively, located on the S protein of PEDV [40]. According to the sequence information for the neutralizing epitope of the transmissible gastroenteritis virus (TGEV), the neutralizing epitope domain COE (aa 499–638) that is responsible for inducing virus neutralizing antibodies on the S1 domain of PEDV S protein was identified. It is highly conserved in PEDV strains [17], [41]. A novel antigenic domain -1368GPRLQPY1347- motif has excellent immunogenicity has been identified on the C-terminal portion of the S protein, which elicited a strong antibody response and induced neutralizing antibodies [5].

For the protection of neonatal and older animals from disease, vaccination is an effective prevention measure. Although, there are many commercial vaccines, the traditional inactivated vaccines have many deficiencies. Therefore, the two diseases are still major problems in the swine industry [3], [16], [27]. The S protein is considered to be a primary target antigen for developing an effective vaccine against coronaviruses. There are no reports comparing the efficiency of S and S1-based vaccines against PEDV; therefore, we evaluated a combined vaccination approach based on the plasmid-driven expression of two proteins, the TGEV S protein and the PEDV S or S1 proteins using a mouse model. This investigation is a first step for development of bivalent vaccines against TGE and PED.

## Materials and Methods

All animal experiments are approved by the Ethics Committee of Northeast Agricultural University within the contract frame from Program for New Century Excellent Talents in University of Ministry of Education of P.R. China (NCET-10-0144).

### Construction of plasmids containing genes encoding TGEV-S1, PEDV-S1 and PEDV-S

The plasmid pcDNA3.1-TGEV-S containing the full-length S gene of TGEV strain Purdue was constructed in our laboratory by common cloning techniques and used as a template for PCR amplification. A sense primer P1 (5' GGGGGCTAGCCCATGAAAAAACTATTTG 3') and an antisense primer P2 (5' CCCCGAATTCTTAGTTTGTCTAATAA 3') which contain *Nhe*I (P1) and *EcoR*I (P2) restriction enzyme sites (underlined), were used for PCR. The plasmid Easy-T-S containing the full-length S gene of PEDV strain CV777 was constructed in our laboratory by common cloning techniques and used as a template for PCR amplification. A sense primer P3 (5' GGGGGTCGACATGGATGTCACTAGGTGCC 3'), two antisense primers P4 (5'CCCCGCGGCCGCTCAAATACTCATACTAAA 3') and P5 (5' CCCCGCGGCCGCTCATCTCTGCACGTGGAC 3') which contain *Sal*I (P3) and *Not*I (P4 and P5) restriction enzyme sites (underlined), were used for PCR.

**Figure 1:**
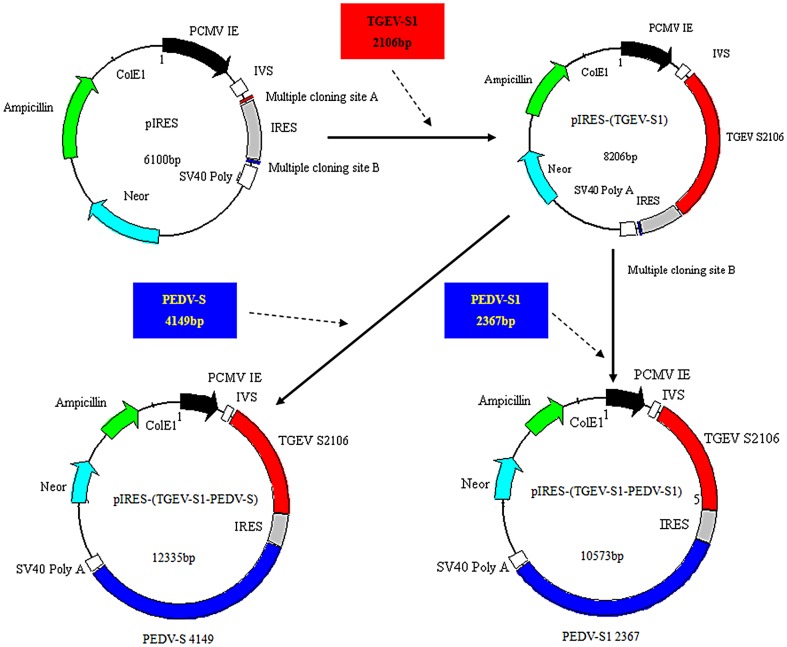
Schematic drawing of construction of DNA plasmids. DNA plasmids. Plasmids pIRES-(TGEV-S1-PEDV-S1) and pIRES-(TGEV-S1-PEDV-S) were generated according to the steps indicated by the arrows.

The amplification profile for the TGEV S1 gene (primers P1/P2) was as follows: 95°C for 5 min followed by 30 cycles of 94°C for 1 min, 54.4°C for 1 min, and 72°C for 3 min. A final extension of 72°C for 10 min was performed at the end of the cycling period. The amplification profile for PEDV-S1 gene (primers P3/P4) and PEDV-S gene (primers P3/P5) were the same as for the TGEV-S1 gene except that the annealing temperature was raised to 56.8°C and 56.4°C, respectively. All PCR products were purified prior to cloning into the eukaryotic expression vector pIRES (TaKaRa, Japan). Briefly, TGEV-S1 was digested with *Nhe*I and *EcoR*I and then inserted into the multiple clone site (MCS) A of the pIRES vector, resulting in a recombinant plasmid pIRES-TGEV-S1; PEDV-S1 and PEDV-S were digested with *Sal*I and *Not*I and then inserted into MCS B of pIRES-TGEV-S1, respectively. The resulting recombinant plasmids were designated as pIRES-(TGEV-S1-PEDV-S1) and pIRES-(TGEV-S1-PEDV-S), respectively. The cloning steps are shown in Figure 1. After transformation, clones were picked and validated by DNA sequencing.

TGEV S1 and PEDV S1 proteins were expressed in *E.coli*, respectively for lymphocyte stimulation assays. In brief, two recombinant plasmids pGEX-S1 comprising the 5′-end half of the TGEV S gene [26] and pET30a-S1 comprising 5′-end of PEDV S gene (97 bp-1776 bp) were constructed and transformed into E.coli BL21 (DE3) pLysS (Novagen, Germany). Expression and purification of TGEV S1 protein were detailed in a recent reference [26] and PEDV S1 protein was also obtained according to a similar protocol. The expressed fused proteins were named TGEV-S1and PEDV-S1, respectively,

### Preparation of plasmid DNA and Western blot of transformed BHK-21 cells

Plasmid DNAs pIRES-(TGEV-S1-PEDV-S1), pIRES-(TGEV-S1-PEDV-S) and pIRES were chromatographically purified from bacterial lysates (Qiagen, Germany), precipitated, and then washed with ethanol. All plasmids were dissolved in 0.1 M PBS to a final concentration of 1 µg/µl. For immunofluorescence assays, BHK-21 cells from American Type Culture Collection (ATCC) were cultured to 90% confluency in 6-well plates at 37°C (approx 24 h) then transformed with 3 µg of pIRES-(TGEV-S1-PEDV-S1) and pIRES-(TGEV-S1-PEDV-S), or pIRES using lipofectamine 2000. The next day, indirect immunofluorescence assays were performed with modifications as described [22], [24], [37]. After fixation by formalin, the cells were incubated for 20 min with 0.2% Triton X-100. Followed by incubation for 1 h with polyclonal antisera (1∶200 dilution in 1% BSA) against PEDV (prepared in our laboratory) or TGEV (prepared in our laboratory). After washing with PBS, the cells were incubated in the dark with FITC-labeled goat anti-rabbit IgG (1∶500) for 1 h. The green fluorescence signals were analyzed by fluorescence microscopy (Leica, Germany). For Western blot, 6 µg of each plasmid were transformed into BHK-21 cells in 6-well plates. BHK-21 cells transfected with empty vector were used as control. After 24 hours, cells were treated with 1% Triton X-100 and centrifuged at 12,000 rpm for 5 minutes, respectively. Then the supernatants were subjected to 10% SDS-PAGE. The proteins were electronically transferred to a nitrocellulose (NC) membrane. The NC membrane was blocked overnight at 4°C using 5% non-fat dry milk in PBS-0.05% Tween-20 (PBST) followed by incubation with the anti-TGEV or anti-PEDV serum (1∶300 dilution in PBST) at 37°C for 1 h. The membrane was incubated with horseradish peroxidase (HRP)-conjugated goat anti-rabbit IgG (1∶2000 dilution in PBST) at 37°C for 1 h, after complete washing with PBST. The protein bands were visualized using chemiluminescence reagents (Roche, Switzerland).

### Immunization of mice

All animal studies were preapproved by the Animal Ethics Committee of Northeast Agricultural University, China (approval ID 1155-NCET-005). Prior to DNA vaccination, six-week-old Kunming mice (Harbin Veterinary Research Institute) were separated into 8 groups (Table 1) and injected in the medial vastus muscle with lidocaine hydrochloride (27 ga needle; 50 µl of 0.8% v/v in PBS). After 15 min, the mice received by similar injection, 100 µg of pIRES-(TGEV-S1), pIRES-(PEDV-S1), pIRES-(PEDV-S) pIRES-(TGEV-S1-PEDV-S1) and pIRES-(TGEV-S1-PEDV-S), attenuated vaccine for TGEV and PEDV, pIRES or PBS (100 µg each). The mice were boosted twice, each at 2-week intervals as shown in Table 1.

**Table 1:** Experimental parameters and Cytokine levels in the blood of immunized mice (^−^X_A_−^−^X_B_ ±s).

Group	Number of mice	Vaccine category	Immunizing dose	Number of treatments	IFN-γ(pg/ml)	IL-4(pg/ml)
A	28	PBS	100 µl	3	24.0003±5.7749	14.3706±3.3531
B	28	pIRES	100 µg	3	20.2144±14.9331	19.32303±2.3205
C	28	pIRES-(TGEV-S1-PEDV-S1)	100 µg	3	72.7261±7.3722	1.015353±2.4213
D	28	pIRES-(TGEV-S1-PEDV-S)	100 µg	3	101.4511±18.9877★	30.92861±3.102
E	28	pIRES-(PEDV-S1)	100 µg	3	61.0910±11.1744	2.699454±4.374
F	28	pIRES-(PEDV-S)	100 µg	3	68.2972±13.0695	10.08804±1.047
G	28	pIRES-(TGEV-S1)	100 µg	3	98.0663±6.4839	46.94724±0.79
H	28	Attenuated TGEV/PEDV vaccine	100 µl	3	139.3929±0.0126	52.17033±3.027

Mice were immunized with PBS (A), pIRES (B), pIRES-(TGEV-S1-PEDV-S1) (C), pIRES-(TGEV-S1-PEDV-S) (D), pIRES-(PEDV-S1) (E), pIRES-(PEDV-S) (F), pIRES-(TGEV-S1) (G) or Attenuated TGEV/PEDV vaccine (H). The number of mice, DNA dosage, and number of immunizations are indicated. The levels of IFN-γ and IL-4 in the serum of immunized mice were analyzed at 42 dpi using commercially available ELISA kits. ★: p<0.01 (highly significant), compared with PBS, pIRES groups.

### Antibody detection and T lymphocyte analysis

Peripheral blood was collected by orbital bleeds at 3 h and then at 7, 14, 21, 28, 35 and 42 days post-immunization (dpi); serum and T lymphocytes were subsequently prepared. For detecting TGEV and PEDV specific antibodies, recombinant TGEV-S1 and PEDV-S1 protein purified from pGEX-(TGEV-S1) and pET-(PEDV-S1) transformed bacterial cells was diluted to 50 µg/ml in 0.05 M NaHCO_3_. An ELISA was performed according to published protocols with modifications [22], [24]. To assess antibody binding, ELISA wells were incubated with o-phenylenediamine dihydrochloride substrate for 15 min. The reactions were terminated with 50 µl 2M H_2_SO_4_ and the wells read at 490 nm.

Peripheral blood lymphocytes were purified using lymphocyte separation solution (Invitrogen, USA) according to the manufacturer's instructions. Cells were suspended to 1×10^7^ cells/ml in RPMI 1640 medium containing 10% serum and further prepared as described [22]. Prepared cells were then incubated at 4°C with FITC-conjugated anti-CD4^+^ T cell antibody or PE-conjugated anti-CD8^+^ T cell antibody (1∶1000 dilutions) (Zhongshan, China) for 30 min. After incubation, the cells were washed with cold PBS (3X), suspended in PBS and subjected to flow cytometry. Splenic lymphocytes were similarly prepared and analyzed.

### Proliferation of T lymphocytes from immunized mice

Splenocytes from immunized mice were prepared for lymphocyte proliferation assays as described [22]. Prepared cells (50 µl of 2×10^6^ cells/ml) were suspended in RPMI1640 containing 10% serum then transferred to 96-well, flat-bottom plates. To each well, 50 µl of medium containing either 20 µg/ml purified recombinant TGEV-S1 and PEDV-S1 proteins or Concanavalin A (Sigma) was added; all treatments were performed in triplicate. Plates were incubated for 72 h, supplemented with 10 µl/well of 3-(4,5-dimethylthylthiazol-2-yl)-2,5-diphenyltetrazoliumbromide (MTT), then incubated for an additional 4 h. Reactions were terminated by adding an equal volume of DMSO and incubating the plates at room temperature for 10 min. Proliferation was measured by OD_490_ values. Proliferation of T lymphocytes in PBMCs from vaccinated and control mice was similarly monitored.

### Neutralization of PEDV and TGEV with immune mouse sera

To determine if mice generated TGEV and PEDV neutralizing antibodies, sera (1∶20–1∶320 dilution, 300 µl) from DNA-vaccinated mice were mixed with an equal volume of PEDV or TGEV (10^5^ pfu/ml) at 37°C. After 1 h incubation, the treated viruses were used to infect cultured Swine testis (ST) Cells and African green monkey kidney (Vero) cells in 24-well plates respectively followed by overlaying with 1% methylcellulose. The plates were incubated at 37°C in a 5% CO_2_ atmosphere and examined daily for 3 days for TGEV and PEDV specific cytopathic effects (CPE).

### Indirect detection of IFN-γ

Serum IFN-γ levels were analyzed with an IFN-γ detection kit (Excell Bio., China) according to the manufacturer's instructions. A standard curve was generated using control IFN-γ diluted in PBS at different concentrations beginning with 10,000 pg/ml followed by two-fold serial dilutions between 2000 pg/ml and 61.25 pg/ml. Dilutions were subsequently coated onto ELISA plates overnight at 37°C. Sera (1∶100) from 42 dpi mice were also coated onto ELISA plates and used as primary antibodies in a parallel experiment to evaluate the virus-derived IFN-γ response. HRP-conjugated goat-anti mouse (1∶2000) was used as secondary antibody in both the control experiment and in the analysis of the mouse sera. The OD_490_ values and therefore pg/ml of IFN-γ in immunized mice were determined relative to the IFN-γ standard curve.

Serum IL-4 levels were similarly analyzed using an IL-4 detection kit (Excell Bio. China). Control IL-4 was serially-diluted two-fold in PBS between 500 pg/ml and 7.8 pg/ml then coated onto ELISA plates at 37°C overnight. The ELISA was performed as above and OD_490_ values (pg/ml) were determined relative to an IL-4 standard curve.

### Cytotoxicity (CTL) assay

Cytotoxicity was analyzed using a lactate dehydrogenase (LDH) release assay kit according to the manufacturer's instructions (Jiancheng, Nanjing, China) using splenic lymphocytes and blood from 42 dpi mice. Lymphocytes, suspended in complete RPMI 1640 medium were used as effector cells. Simultaneously, target ST and Vero cells were infected with TGEV and PEDV, respectively, at a titer of 100TCID_50_ for 36 h at 37°C under 5% CO_2_. The effector cells were mixed with the sensitized target cells at 25∶1, added to each well of a 96-well round-bottom microplate, and incubated for 6 h at 37°C. After centrifugation at 1500 rpm for 10 min, 100 µl of supernatant was collected and transferred to a fresh 96-well flat-bottomed plate, followed by the addition of 100 µl/well of LDH assay reagent. The mixture was allowed to incubate for 15 min at 37°C after which the OD_490_ was measured. Spontaneous release of LDH was determined using samples prepared from target cells cultured in medium alone; maximum LDH release was measured using samples prepared by lysis of target cells in medium containing 1% (v/v) Triton X-100. The CTL was calculated using the following equation: [(experimental release-spontaneous release)/(maximum release-spontaneous release)] x 100. All experiments were performed in triplicate.

### Statistical analysis

Statistical analysis of the data was performed using SPSS 11.5 software; p<0.05 and p<0.01 were defined as statistically significant and statistically very significant, respectively.

## Results

### In vitro expression of pIRES-(TGEV-S1-PEDV-S1) and pIRES-(TGEV-S1-PEDV-S)

**Figure 2:**
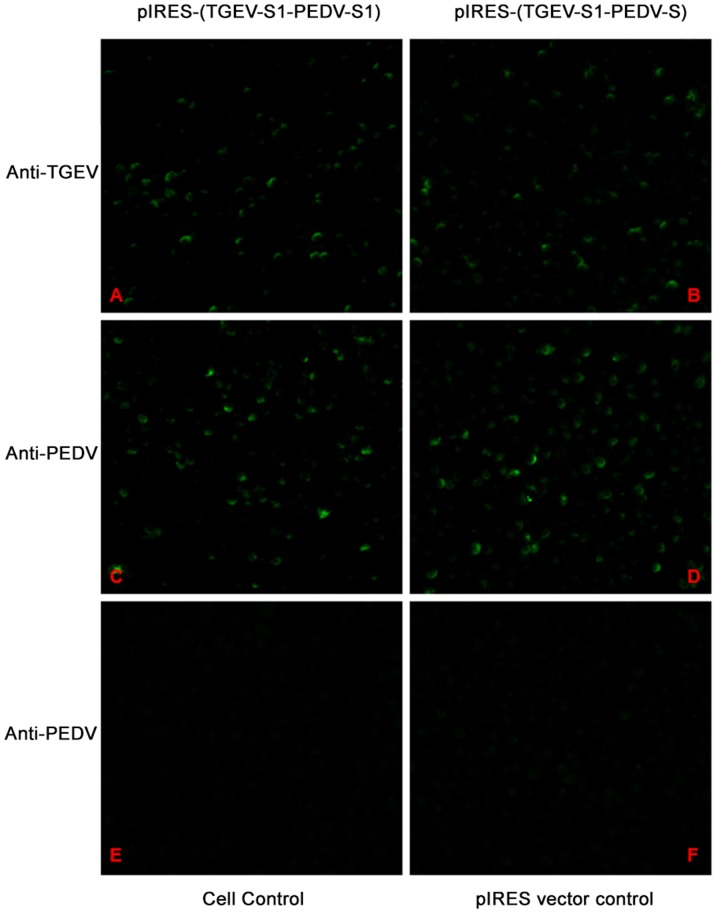
Immunofluorescence analysis of cells transfected with recombinant plasmids. BHK-21 cells were transfected with pIRES-(TGEV-S1-PEDV-S1) or pIRES-(TGEV-S1-PEDV-S). Transient expression of proteins within the transfected cells was detected with anti-PEDV antibodies and anti-TGEV antibody respectively. The green signals reflect expression of the respective proteins of interest. (A) and (B): Cells transfected with pIRES-(TGEV-S1-PEDV-S1) and pIRES-(TGEV-S1-PEDV-S); transiently expressed TGEV S1 protein was detected by anti-TGEV antibody; (C) and (D): transient expression of PEDV S protein was detected by anti-PEDV antibody; (E): cell control; (F): pIRES vector control. Samples in E and F were stained by anti-PEDV. The same pictures were obtained when control cells were stained by anti -TGEV (not shown).

**Figure 3:**
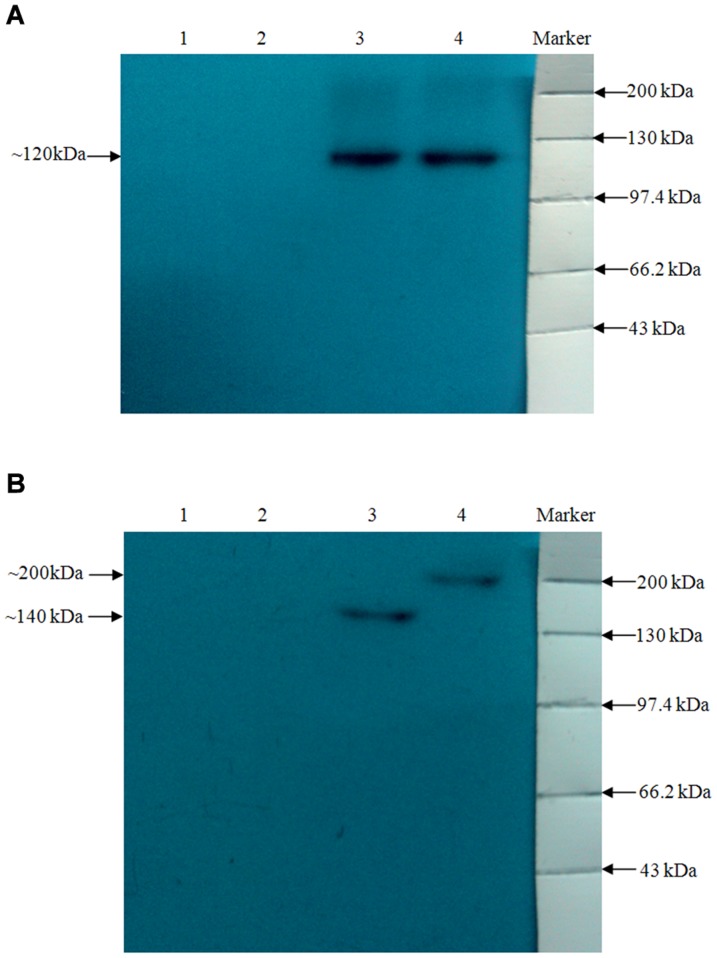
Western blot of cells transfected with recombinant plasmids. BHK-21 cells were transfected with pIRES-(TGEV-S1-PEDV-S1) or pIRES-(TGEV-S1-PEDV-S). Transient expression of proteins within the transfected cells was detected by Western blot with anti-TGEV antibodies (Panel A) and anti-PEDV antibody (Panel B), respectively. Panel A; (1): cell control; (2): pIRES vector control. (3): Cells transfected with pIRES-(TGEV-S1-PEDV-S1) and (4): Cells transfected with pIRES-(TGEV-S1-PEDV-S). Panel B; (1): cell control; (2): pIRES vector control. (3): Cells transfected with pIRES-(TGEV-S1-PEDV-S1) and (4): Cells transfected with pIRES-(TGEV-S1-PEDV-S).

Sequence analysis of pIRES-(TGEV-S1-PEDV-S1) and pIRES-(TGEV-S1-PEDV-S) showed 100% identity of the S sequences compared to those of GenBank accession numbers DQ811789.1 (TGEV-S1) and AF353511.1 (PEDV-S1 and S), respectively. Analysis by immunofluorescence microscopy showed that transfection of BHK-21 cells with pIRES-(TGEV-S1-PEDV-S1) and pIRES-(TGEV-S1-PEDV-S) plasmids resulted in the expression of the S or S1 proteins, respectively, of TGEV and PEDV (Figure 2). Western blot further confirmed the in vitro expression of these genes of interest (Figure 3).

### T lymphocyte proliferation in spleen and blood

**Figure 4:**
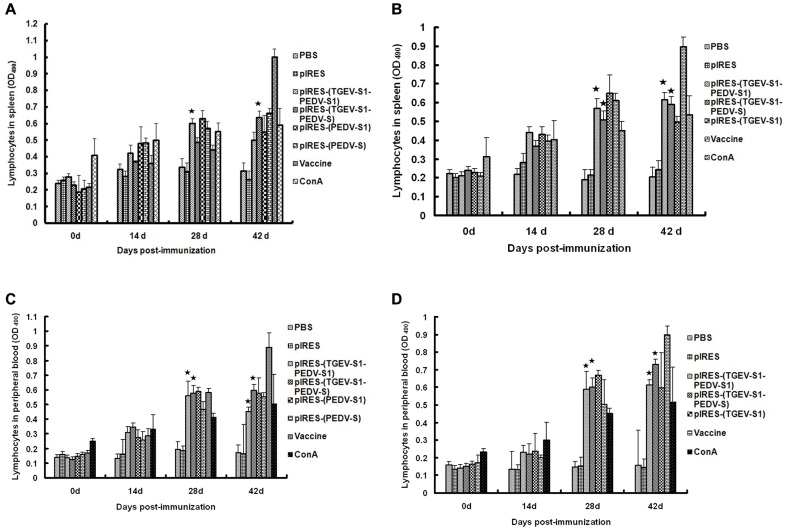
Changes in T lymphocyte numbers in the spleens and peripheral blood of immunized mice. The proliferation of T lymphocytes in mouse spleens (A and B) and peripheral blood (C and D) was analyzed by conventional MTT assays using recombinant PEDV-S1 (A and C), TGEV-S1 (B and D) protein and Con A as stimulating agents. The y-axis represents the lymphocyte proliferation index (OD_490_) in the spleen or peripheral blood. ★: p<0.01 (highly significant) compared with PBS group and pIRES group.

To determine the immune response of mice to the DNA vaccination, plasmids were applied by intramuscular injection. At different times after vaccination, animals were sacrificed and analyzed for various parameters of the immune reaction. The proliferation of spleen T lymphocytes upon stimulation with purified S1 protein from either TGEV or PEDV was analyzed by MTT assays. As shown in Figure 4A, when stimulated with PEDV-S1 protein at 28 dpi, the proliferation levels of spleen T lymphocytes from mice injected with pIRES-(TGEV-S1-PEDV-S1) and pIRES-(PEDV-S1) were increased compared to those of control cells and the increase was highly significant (p<0.01). By 42 dpi, the proliferation levels of cells from mice injected with pIRES-(TGEV-S1-PEDV-S1) or pIRES-(PEDV-S1) were somewhat lower but still were significantly increased when compared to the control cells (p<0.01). By contrast, the proliferation levels of lymphocytes from animals immunized with pIRES-(TGEV-S1-PEDV-S) or pIRES-(PEDV-S) were highest at 42 dpi (p<0.01); no significant differences (p>0.05) were observed between the groups of pIRES-(TGEV-S1-PEDV-S) and pIRES-(PEDV-S). The proliferation values upon stimulation by TGEV-S1 protein are shown in Figure 4B. By 28 dpi the proliferation levels of spleen T lymphocytes from animals injected with pIRES-(TGEV-S1-PEDV-S1), pIRES-(TGEV-S1-PEDV-S) or pIRES-(TGEV-S1) were all significantly increased (p<0.01) relative to the PBS or pIRES controls. Furthermore, the values of the pIRES-(TGEV-S1-PEDV-S1) group were somewhat higher than those of the pIRES-(TGEV-S1-PEDV-S) group (p<0.05). By 42 dpi, the changes in the proliferation levels relative to controls remained highly significant (p<0.01) irrespective of the plasmid used for vaccination, pIRES-(TGEV-S1-PEDV-S1) or pIRES-(TGEV-S1-PEDV-S). No significant differences (p>0.05) were observed between the 28 dpi and 42 dpi values. Assays using PBMC to determine the proliferation of peripheral blood lymphocytes upon stimulation by PEDV-S1 protein were performed. At 14 dpi the proliferation of blood lymphocytes from mice immunized with pIRES-(TGEV-S1-PEDV-S1) or pIRES-(TGEV-S1-PEDV-S) was significantly increased (p<0.05) relative to the PBS and pIRES controls (Figure 4C) and this difference remained significant (p<0.01) when cells were analyzed at 42 dpi. The values of the two time points were similar in the case of the mice immunized with pIRES-(TGEV-S1-PEDV-S). In the pIRES-(TGEV-S1-PEDV-S1) group, the proliferation values at 42 dpi were slightly but significantly lower (p<0.05). As shown in Figure 4D, when stimulated with TGEV-S1 protein, the proliferation of PBMC was increased at 28 and 42 dpi in mice immunized with pIRES-(TGEV-S1-PEDV-S1) and pIRES-(TGEV-S1-PEDV-S) (p<0.01) relative to PBS and pIRES controls. In the group pIRES-(TGEV-S1-PEDV-S), the proliferation significantly increased from 28 to 42 dpi whereas the proliferation of PBMC from mice immunized with pIRES-(TGEV-S1-PEDV-S1) was similar at both time points.

### Changes in CD4+ and CD8+ T lymphocytes

**Figure 5:**
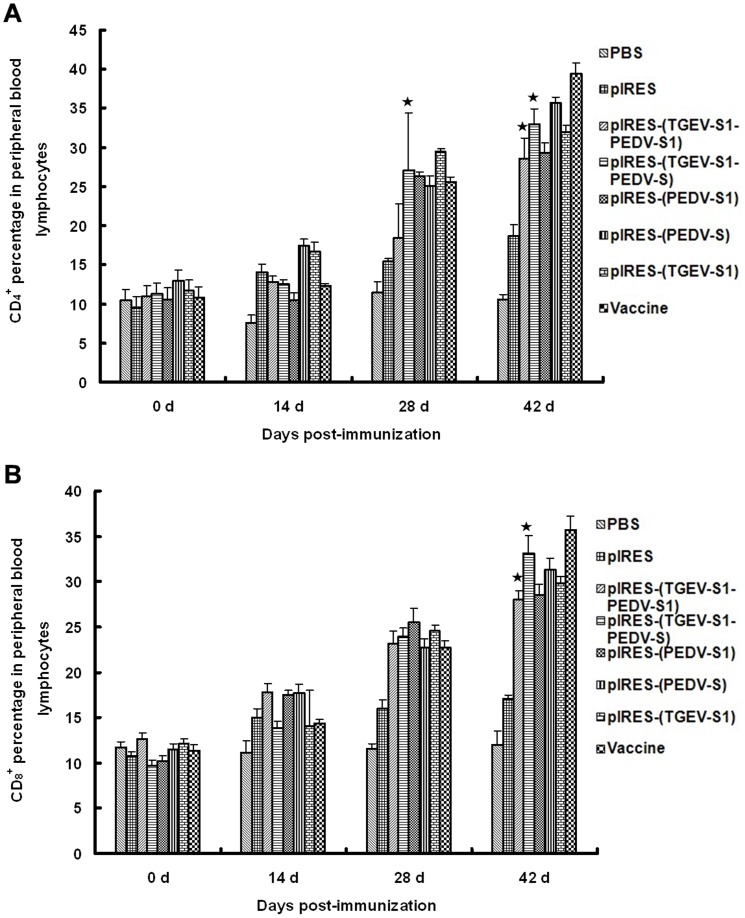
Changes in CD_4_^+^ and CD_8_^+^ T lymphocytes in the peripheral blood of immunized mice. Lymphocytes from the peripheral blood of mice treated with recombinant plasmids were collected and subjected to flow cytometry to assess the numbers of CD_4_^+^ T lymphocytes (A) and CD_8_^+^ T lymphocytes (B). ★: p<0.01 (highly significant), compared with PBS group and pIRES group.

**Figure 6:**
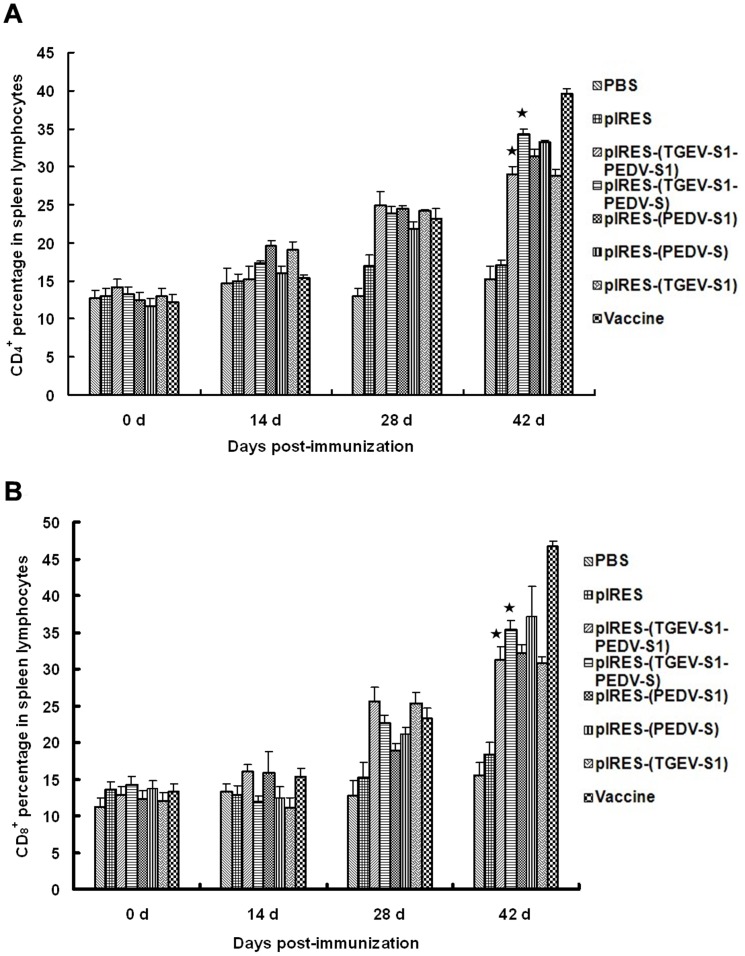
Changes in CD_4_^+^ and CD_8_^+^ T lymphocytes in the spleens of immunized mice. Spleen cells from mice immunized with recombinant plasmids were collected and subjected to flow cytometry to assess the numbers of CD_4_^+^ T lymphocytes (A) and CD_8_^+^ T lymphocytes (B). ★: p<0.01 (highly significant), compared with the PBS and pIRES groups.

Flow cytometry data showed that the levels of CD_4_ *^+^* and CD_8_ *^+^* T lymphocytes in peripheral blood gradually increased from 14 dpi to 42 dpi (Figure 3). The levels of CD_4_ *^+^* cells (Figure 5A) in mice immunized with pIRES-(TGEV-S1-PEDV-S) was significantly (p<0.01) increased already at 28 dpi. By 42 dpi the number of CD_4_ *^+^* T cells from mice immunized with pIRES-(TGEV-S1-PEDV-S1) was also increased but the value for mice immunized with pIRES-(TGEV-S1-PEDV-S) was higher. In the case of CD_8_ *^+^* cells, cell numbers were increased at 28 dpi in mice immunized with pIRES-(TGEV-S1-PEDV-S1) and pIRES-(TGEV-S1-PEDV-S) to a similar extent (Figure 5B). A further increase by 42 dpi was observed in the pIRES-(TGEV-S1-PEDV-S) group (p<0.05) whereas in the pIRES-(TGEV-S1-PEDV-S1) group at 42 dpi only a slight increase was determined. When spleen CD_4_ *^+^* lymphocyte numbers were examined by flow cytometry, the results again showed a gradual increase from 14 dpi to 42 dpi (Figure 6A), and the highest value was determined for the pIRES-(TGEV-S1-PEDV-S) group at 42 dpi. Analyses of spleen-derived CD_8_ *^+^* T cells (Figure 6B) in animals immunized with pIRES-(TGEV-S1-PEDV-S1) or pIRES-(TGEV-S1-PEDV-S) showed a clear increase of the cell numbers by 28 and 42 dpi.

### Antibody detection in immunized mice

**Figure 7:**
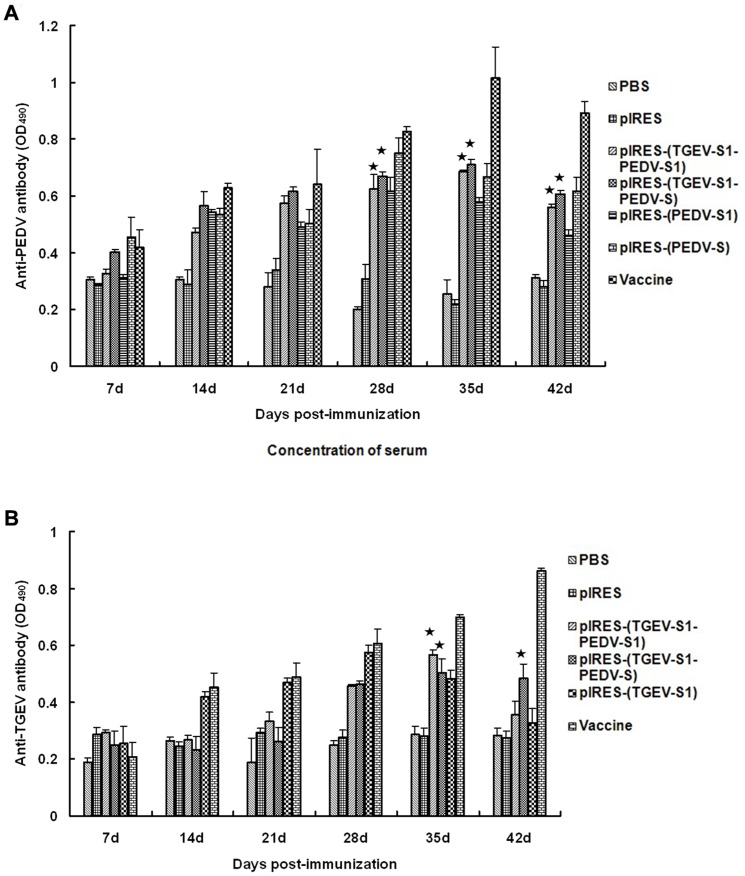
Antibody levels in mice treated with pIRES-(TGEV-S1-PEDV-S1) and pIRES-(TGEV-S1-PEDV-S). Anti-PEDV serum antibodies (A) and anti-TGEV serum antibodies (B) in pIRES-(TGEV-S1-PEDV-S1) and pIRES-(TGEV-S1-PEDV-S) immunized mice were detected by indirect ELISA at different time points following the injection of the plasmid DNAs. The OD_490_ was monitored as a function of time over a period of 42 days. ★: p<0.01 (highly significant), compared with PBS, pIRES treatment groups.

Serum antibody levels against PEDV and TGEV were examined in mice immunized with plasmid DNA using an indirect ELISA. In general, between 14-42 dpi, the levels of PEDV antibodies in mice immunized with pIRES-(TGEV-S1-PEDV-S1) and pIRES-(TGEV-S1-PEDV-S) were similar and they were significantly (p<0.01) higher than those from animals injected with PBS or empty vector (Figure 7A). As shown in Figure 7B, the level of TGEV antibodies began to increase at 28 dpi and reached the peak at 35 dpi. Between 35–42 dpi, the antibody levels decreased significantly (p<0.01) in the pIRES-(TGEV-S1-PEDV-S1) group, but not in the pIRES-(TGEV-S1-PEDV-S) group.

### Neutralizing antibodies

**Figure 8:**
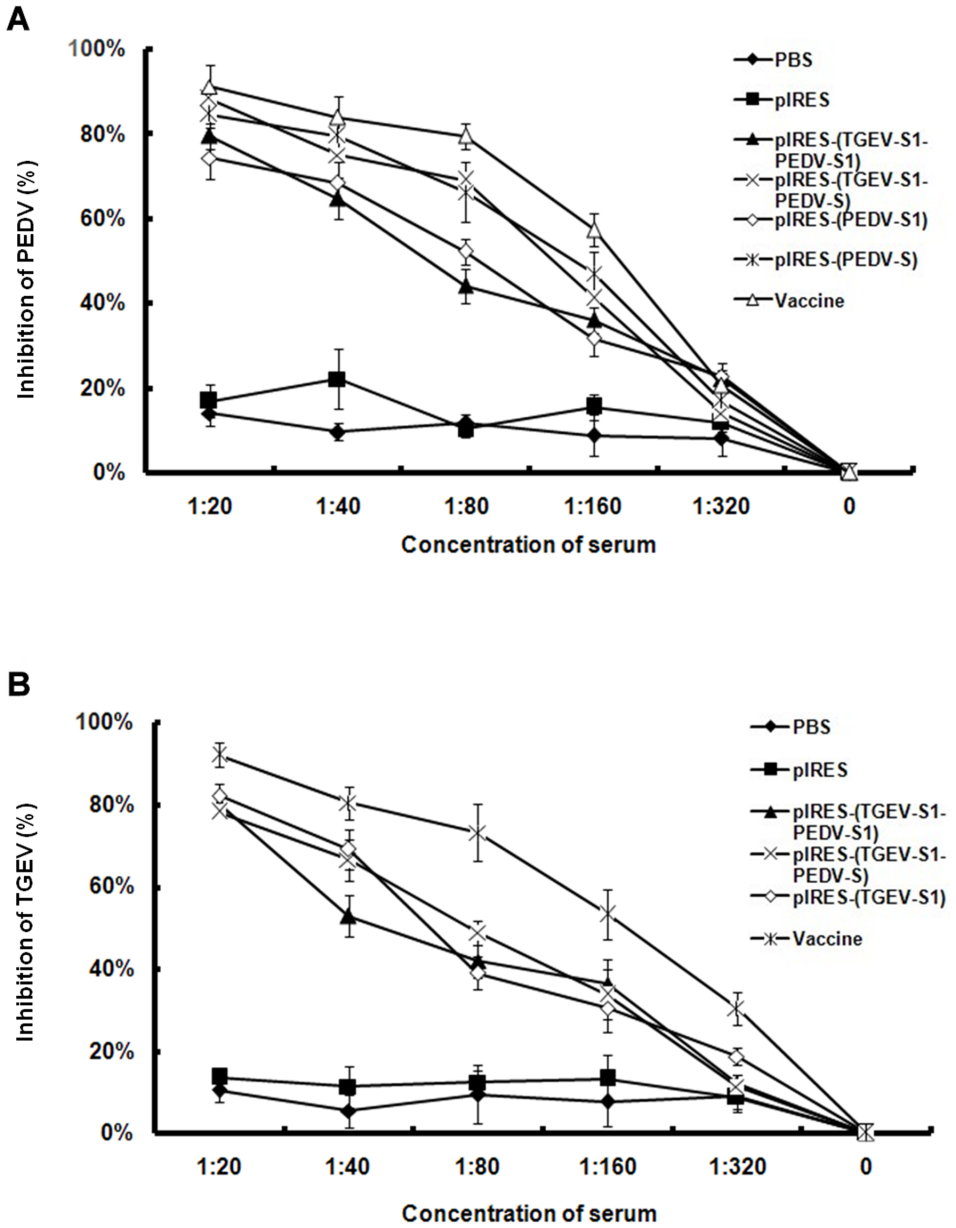
Levels of neutralizing antibodies in mice treated with pIRES-(TGEV-S1-PEDV-S1) and pIRES-(TGEV-S1-PEDV-S). Anti-PEDV and anti-TGEV neutralizing antibodies in immunized mice were detected by plaque reduction assay with different serum dilutions from blood samples taken at 28 days after the injection of the plasmid DNAs.

Assays for neutralizing anibodies against PEDV (Figure 8A) showed a concentration-dependent decrease of the inhibitory activity in sera of mice immunized by pIRES-(PEDV-S1), pIRES-(PEDV-S), pIRES-(TGEV-S1-PEDV-S1) and pIRES-(TGEV-S1-PEDV-S); higher titers were determined in sera from mice immunized with pIRES-(PEDV-S) and pIRES-(TGEV-S1-PEDV-S) constructs as compared to animals injected with pIRES-(PEDV-S1) and pIRES-(TGEV-S1-PEDV-S1) constructs. Neutralizing antibodies against TGEV (Figure 8B) were also detected in mice immunized by pIRES-(TGEV-S1), pIRES-(TGEV-S1-PEDV-S1) and pIRES-(TGEV-S1-PEDV-S). Here, there were no pronounced differences detected between the three groups of animals.

### Changes in the levels of serum IFN-γ and IL- 4 in immunized mice

Changes in serum IFN-γ and IL-4 levels in immunized mice were analyzed using ELISA. The results showed that the levels of IFN-γ in mice treated with pIRES-(PEDV-S1), pIRES-(PEDV-S), pIRES-(TGEV-S1), pIRES-(TGEV-S1-PEDV-S1), pIRES-(TGEV-S1-PEDV-S) and Vaccine were significantly higher than in mice treated with PBS or pIRES. The highest level of IFN-γ was found to be induced by pIRES-(TGEV-S1-PEDV-S) (p<0.05) (Table 1). However, the levels of IL-4 in all treatment groups were fluctuant, especially, in pIRES-(TGEV-S1-PEDV-S1) and pIRES-(PEDV-S1) group which did not induce any IL4 production and the IL-4 level even much lower (P<0.05) than PBS and pIRES control. The pIRES-(TGEV-S1) group was higher (P<0.05) than pIRES-(TGEV-S1-PEDV-S), and both are highly significant (P<0.01) than PBS and pIRES control.

### Activity of CTL in spleen and blood

**Figure 9:**
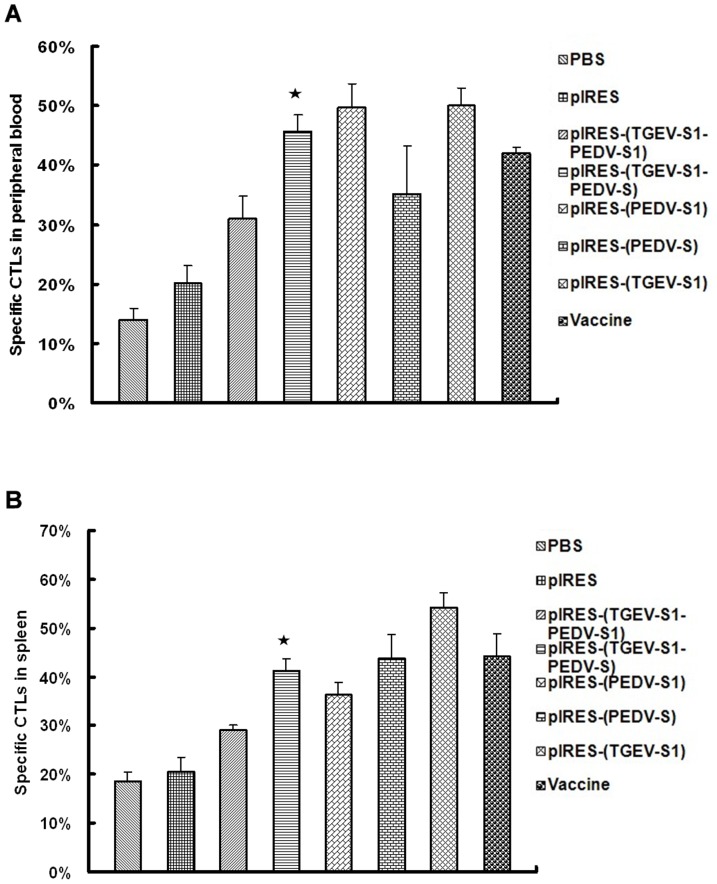
Activity of CTLs in spleen and peripheral blood. Cytotoxicity was analyzed using the LDH release assay. The release of LDH which is directly proportional to CTL activity was monitored as a function of time over a period of 42 days. ★: p<0.01 (highly significant) compared with PBS, pIRES treatment groups.

Cytotoxicity was analyzed using the LDH release assay. The results showed that CTL function in the peripheral blood of the mice treated with pIRES-(TGEV-S1-PEDV-S1) was higher (P<0.05) than in control cells, but significantly lower than the values determined in the pIRES-(PEDV-S1) and pIRES-(TGEV-S1-PEDV-S) groups (p<0.01). In addition, the CTL function of pIRES-(TGEV-S1-PEDV-S) group was higher (P<0.05) than that of the pIRES-(PEDV-S) group, (Figure 9A). In a similar way, the CTL function of spleen cells was determined for animals immunized by pIRES-(PEDV-S1), pIRES-(PEDV-S), pIRES-(TGEV-S1), pIRES-(TGEV-S1-PEDV-S1), pIRES-(TGEV-S1-PEDV-S) and Vaccine. In all cases the values were significantly increased compared to the control cells (Figure 9B).

## Discussion

Prevalence of TGE and PED may lead to severe economic losses in swine-raising countries. So far, the most effective prevention measure is vaccination. DNA vaccines can induce complete immune responses, provide heterologous cross protection and can be easily prepared as polyvalent vaccines [23]. It is documented that the S1domain of TGEV/PEDV contains the main neutralizing epitopes [13], [20], [34], [39]. One report indicated that the immunogenicity of DNA plasmid bearing TGEV S1 gene is superior to another version containing the full-length S gene [29]. In contrast, there are no reports regarding comparative analysis on the efficiency of S and S1-based DNA plasmids. Therefore, this study aimed at evaluating immune response induced by DNA plasmids encoding the TGEV S protein and the PEDV S or S1 proteins using a mouse model. In pIRES vector, there is an internal ribosomal entry site (IRES), which is derived from the encephalomyocarditis virus (ECMV); two multiple cloning sites (MCS) A and B, allow each product of transcription to be translated independently with the participation of ribosome at the same time. In addition, pIRES vector has a CMV promoter for high expression of foreign genes and a SV40 enhancer/promoter that allows the enhancement of gene expression in many hosts [33]. Therefore, the genes of interest in this study were cloned into the pIRES vector. Both immunufluorescence assays and Western blot indicated that the *in vitro* expression of TGEV S, PEDV S or PEDV S1 proteins were successful.

It is clear that the level of neutralizing antibodies is an important indicator to evaluate the effect of the vaccine. In this study, IgG levels of PEDV antibodies in pIRES-(TGEV-S1-PEDV-S1) and pIRES-(TGEV-S1-PEDV-S) groups increased between 21–42 dpi, and the differences between both groups were not significant. In addition, the values of the pIRES-(TGEV-S1-PEDV-S1) and pIRES-(TGEV-S1-PEDV-S) groups peaked at 35 dpi and decreased thereafter, but the differences between these two groups were also not significant. The IgG antibody levels against TGEV of these two groups began to increase at 28 dpi and reached the peak at 35 dpi. Between 35–42 dpi, the antibody levels decreased dramatically (p<0.01) in pIRES-(TGEV-S1-PEDV-S1) group, whereas no significant changes was observed (p>0.05) in the pIRES-(TGEV-S1-PEDV-S) group. This result showed that the recombinant plasmid pIRES-(TGEV-S1-PEDV-S) containing the full-length S protein of PDEV was better in eliciting immune responses to PEDV than is pIRES-(TGEV-S1-PEDV-S1) encoding only the S1 portion of the S protein. The IgG antibody levels and virus neutralizing assays showed that both pIRES-(TGEV-S1-PEDV-S1) and pIRES-(TGEV-S1-PEDV-S) groups could elicit humoral immune responses against PEDV and TGEV S proteins, respectively. Plasmid pIRES-(TGEV-S1-PEDV-S) was more efficient in inducing neutralizing antibodies than pIRES-(TGEV-S1-PEDV-S1).

T cells are important effector cells of immune responses. Their activation causes the secretion of cytokines that advance cellular and humoral immune responses and mediate CTL activity. As such, monitoring CTL activity provides a good readout for immune stimulation. In this study, spleen and blood-derived lymphocytes from immunized mice clearly showed that immunization with pIRES-(TGEV-S1-PEDV-S1) and pIRES-(TGEV-S1-PEDV-S) significantly induced T cell proliferation. When stimulated with purified recombinant PEDV-S1 protein, there was higher T lymphocyte proliferation in the pIRES-(TGEV-S1-PEDV-S) group than in the pIRES-(TGEV-S1-PEDV-S1) group.

Most exogenous proteins and even inactivated pathogens are internalized and processed via the endolysosomal pathway; the resulting degradation of proteins yields peptides that are associated with MHC-II molecules. They not only may stimulate antibody responses but also the production of helper T cells, which are necessary for the specificity of antibodies and CTLs production. Activated CD_4_^+^ T cells can be classified into at least two subgroups, Th1 and Th2. The Th1-like phenotype is predominantly associated with interleukin-2 (IL-2) and interferon-γ (IFN-γ) [30], [43]–[46]; The Th2-like phenotype, characterized by increased levels of interleukin-4 (IL-4), interleukin-5 (IL-5), and interleukin-6 (IL-6), is associated with improving humoral response and mucosal immune response [21], [38]. In this study, the number of CD_4_^+^ T lymphocytes in peripheral blood and spleen reached the peak at 42 dpi and the number of CD_4_ *^+^* T cells from mice immunized with pIRES-(TGEV-S1-PEDV-S) was higher (p<0.05) than pIRES-(TGEV-S1-PEDV-S1) group. Changes in serum IFN-γ and IL-4 levels in immunized mice were analyzed. The results showed that the levels of IFN-γ in mice treated with DNA vaccine were significantly higher. In addition, the levels of IFN-γ induced by pIRES-(TGEV-S1-PEDV-S) were higher than other groups (p<0.05). However, the levels of IL-4 in all treatment groups were fluctuant, especially, in pIRES-(TGEV-S1-PEDV-S1) and pIRES-(PEDV-S1) group the IL-4 level even much lower (P<0.05) than PBS and pIRES control. This result may be explained by the high level of IFN-γ promoting the Th1 cell differentiation, at the same time, restraining the Th2 cell differentiation by down-regulating IL-4 and humoral immune response. This tendency is consistent with the IgG antibody changes in 42 dpi. Several reports also suggested that the level of antigen presentation can affect Th1/Th2 profiles [4].

Following an increased understanding of the mechanisms of antigen processing and presentation for the generation of MHC-I restricted cytolytic T-lymphocyte (CTL) responses, the importance of cellular immunity became significant. The reasons for seeking to specifically generate CTLs include not just their activity of directly killing infected cells (versus directly killing the virus or bacteria) but also the fact that CTLs can focus on antigens that are not accessible to antibodies [25]. To elicit CTL responses, the antigen needs to be present in the cytoplasm of antigen-presenting cells (APCs). Then some of the newly synthesized proteins are processed in proteosomes with certain of the resultant peptides then binding to nascent MHC-I molecules for export via the Golgi to the cell surface [25]. Therefore, specific CTL activity could reflect the activity of the CD8+ T lymphocytes. In this study, the number of CD8+ T lymphocytes in peripheral blood and spleen reached the peak at 42 dpi and the number of CD8+ T cells from mice immunized with pIRES-(TGEV-S1-PEDV-S) was higher (p<0.05) than in the pIRES-(TGEV-S1-PEDV-S1) group, similar to the CD4+ T lymphocytes changes. The activity determined by the CTL assay showed that CTL function in the peripheral blood of the mice treated with pIRES-(TGEV-S1-PEDV-S1) was higher (P<0.05) than that of the PBS control, but significantly lower than in the pIRES-(TGEV-S1-PEDV-S) group (p<0.01). As with other studies, CTL functions in spleen cells were mirrored by a higher efficiency of the pIRES-(TGEV-S1-PEDV-S) group compared to the pIRES-(TGEV-S1-PEDV-S1) group.

In summary, to the best of our knowledge, this is the first time that the immunological efficacies triggered by PEDV S protein and the TGEV S protein were compared using a mouse model. Both the recombinant plasmids pIRES-(TGEV-S1-PEDV-S1) and pIRES-(TGEV-S1-PEDV-S) could elicit in mice cellular immunity, T-cell response as well as humoral response generating CTLs, helper T cells, particularly of Th1 cells, as well as specific neutralizing antibodies against PEDV and TGEV, respectively. It has been reported that an antigenic domain in the S gene that could elicit a strong antibody response and induce neutralizing antibodies has excellent immunogenicity on the C-terminal portion of the S protein [5]. Our results confirmed that the full-length PEDV S gene induces a better immune response than the N-terminal half alone.

As mice are not susceptible to infection by TGEV or PEDV we cannot use this animal model to perform challenge experiments and evaluate the protection rate of the DNA constructs. However, we did observe good immune responses of these DNA plasmids in this species. On the basis of this study, our further studies will include usage of pigs to optimize immunization procedures as well as to evaluate host immunity and protection induced by these DNA plasmids to develop effective DNA vaccines for controlling TGE and PED.
